# Transition Care for Young Persons with Rare Bone Mineral Conditions: A Consensus Recommendation from the ECTS Rare Bone Disease Action Group

**DOI:** 10.1007/s00223-025-01382-w

**Published:** 2025-05-09

**Authors:** Corinna Grasemann, Joline Wernsmann, Natasha M. Appelman-Dijkstra, Chloe Morgan, Tenna Toft Sylvest, Adalbert Raimann, Heide Siggelkow, Willem F. Lems, Serap Turan, M. Carola Zillikens, Lena Lande Wekre, Inês Alves, M. Cohen Solal, Maria P. Yavropoulou, Gavin Clunie

**Affiliations:** 1 Department of Pediatrics, European Reference Network On Rare Endocrine Conditions (ENDO ERN) Reference Center Katholisches Klinikum Bochum, Ruhr-University Bochum, Bochum, Germany; 2 Subdivision of Endocrinology, Department of Internal Medicine, Center for Bone Quality, Leiden University Medical Center, Leiden, The Netherlands; 3 College of Human and Health Studies, Swansea University, Swansea, UK; 4 Forenklingsstyrelsen and Arhus University, Kopenhagen, Denmark; 5 Division of Pediatric Pulmonology, Allergology and Endocrinology, Department of Pediatrics and Adolescent Medicine, Medical University of Vienna, Vienna, Austria; 6 Vienna Bone and Growth Center, Vienna, Austria; 7 Department of Trauma, Orthopedics and Reconstructive Surgery, University Medical Center Göttingen, Göttingen, Germany; 8 MVZ Endokrinologikum Göttingen, Göttingen, Germany; 9 Department of Rheumatology, Amsterdam UMC, Amsterdam, The Netherlands; 10 Division of Pediatric Endocrinology, Department of Pediatrics, School of Medicine, Marmara University, Istanbul, Turkey; 11 Department of Internal Medicine, Erasmus MC, Erasmus University Medical Center, Rotterdam, The Netherlands; 12 TRS National Resource Centre for Rare Disorders, Sunnaas Rehabilitation Hospital, 1453 Nesodden, Oslo Norway; 13 Department of Sport and Health, ANDO Portugal, University of Évora-CHRC, Évora, Portugal; 14 Department of Rheumatology, Lariboisière Hospital, Inserm U1132 and Université Paris Cité, Paris, France; 15 First Department of Propaedeutic and Internal Medicine, Endocrinology Unit, National and Kapodistrian University of Athens, LAIKO General Hospital of Athens, Athens, Greece; 16 Cambridge University Hospitals, Hills Road, Box 204, Cambridge, UK

**Keywords:** Transition, Rare bone mineral conditions, Fragility, Rare disease, XLH, OI, Achondroplasia

## Abstract

Transition care (TC) is crucial for young persons with rare bone and mineral conditions (RBMCs) as they move from pediatric to adult healthcare. Effective TC prevents care disruptions and supports medical and psychosocial needs. However, gaps in communication, a shortage of adult RBMC specialists, and challenges in navigating adult healthcare necessitate standardized care. This study aimed to develop consensus-based recommendations for TC in RBMCs, focusing on best practices for seamless transition and patient empowerment. A two-round Delphi survey (September 2023–April 2024) was conducted among European RBMC experts, including 3 pediatric and 8 adult clinicians and 3 patient representatives from the European Calcified Tissue Society (ECTS). The panel formulated and refined statements through literature review and iterative scoring. Statements reaching ≥ 70% consensus were retained. A total of 81 statements were finalized across seven domains: initiation and planning, TC requirements, patient empowerment, organization and communication, service infrastructure and funding, and clinical care. Consensus was achieved on 64 out of 81 statements, with strong agreement on general and RBMC-specific recommendations. Key priorities included structured coordination among healthcare providers and a patient-centered approach that fosters self-advocacy and self-management. This Delphi consensus provides a structured framework for TC in young persons with RBMCs, emphasizing multidisciplinary care and patient empowerment. Future studies should assess the feasibility and impact of these guidelines across diverse healthcare systems.

## Introduction

The transition of young persons with chronic conditions from the pediatric to the adult care setting should be an age-appropriate coordinated process[1]. It is estimated that 10% of all adults who receive care for chronic conditions have been transferred from pediatric care[2]. However, the Transition Care (TC) period often leads to various challenges, such as gaps in communication between the healthcare providers from different specialties and lack of familiarity with the new adult-based healthcare system with the potential for disruption of ongoing treatment resulting in long-term adverse sequalae [3, 4].

A structured TC process (TCP) aims to ensure continuity of care by seamlessly and effectively transferring medical, and other care responsibilities and facilitating an adaptation to the new healthcare system [5, 6].

The TCP for young persons with rare and chronic conditions needs to encompass components specific to enabling uninterrupted and comprehensive personal support, education on self-management skills and empowerment of the young person to advocate for their own healthcare needs [6, 7]. During the transition period, individual support is of particular importance, as it is often difficult to locate experts in the adult care setting for the rare disease and given a relative lack of disease-specific information for patients [8].

According to the European Union definition, rare diseases (RD) are those affecting fewer than 5 in 10,000 individuals [The European Parliament and the Council of the European Union; https://health.ec.europa.eu/rare-diseases-and-european-reference-networks/rare-diseases_en (last accessed 21–01–2025)]. Despite their rarity individually, collectively these diseases impact a significant portion of the population [9].

Within the Group of RDs, the Rare Bone and Mineral Conditions (RBMCs) constitute a diverse group of conditions characterized by abnormalities in bone development, structure, or metabolism [10], including the large and growing group of more than 750 different skeletal dysplasias, calcium-phosphate disorders, and disturbances in growth and mineralization, often leading to an increased risk of fractures, deformities and associated comorbidities. Owing to the complexity, heterogeneity and rarity of RBMCs, young persons with these conditions require highly specialized multidisciplinary care to address both their medical and also developmental and psychosocial needs [11–13].

Different groups have previously tried to define transition needs in different groups of RBMCs [14–17], and have queried relevant caregivers on their views on the process [11, 18]. Here, the European Calcified Tissue Society (ECTS) Rare Bone Disease Action Group presents consensus-based recommendations for the most important aspects of the TCP for young persons with RBMCs. These recommendations represent expert opinion and serve as a first step, pending future empirical validation.

## Methods

### Group Members (Panelists)

A DELPHI consensus survey was developed and scored by the panelists consisting of patient representatives and clinicians from different European countries (Austria, Denmark, France, Germany, Greece, Norway, Portugal, UK, Sweden, The Netherlands and Turkey). Patient representatives for Osteogenesis Imperfecta (CM), Achondroplasia (IA) and X-linked Hypophosphatemia (TT) who were selected via national alliances/ERN structures and clinicians (experts) of different disciplines (for details see below) from the Rare Bone Disease Action Group of the ECTS were included.

Clinicians had either been trained as adult rheumatologists (GC, WL, MCS), pediatric endocrinologists (CG, AR, ST), adult endocrinologists (MCZ, HS, MPY, NAD) or rehabilitation specialists (LLW) and were all recognized specialists in rare bone mineral conditions with longstanding experience. ERN BOND guideline representation (LLW) and ENDO ERN MTG2 representation (CG) ensured the connection to the ERNs most involved in RBMCs (however due to the multidisciplinary nature of RBMCs other ERNs are also involved, e.g. ERKNET, MetabERN).

The survey was conducted between September 2023 and April 2024.

### DELPHI Methodology

In this study a conventional, digital DELPHI methodology using Calibrum Tool analysis (Calibrum International, Virginia, USA) was followed [19]. Briefly, the methodology was: (1) formation of expert panel (including patient representation, pediatric and adult specialists in different areas of RBMCs); (2) a small (‘core’) group of panel members to screen the literature and generate statements for the Delphi Process; (3) core panel members to discuss statements to identify duplicates and merge similar statements; (4) to define ‘consensus’ at 70% agreement for statement survival based on prior publications regarding DELPHI processes [19, 20]; (5) the resulting statements to be scored using a digital DELPHI (eDelphi) process by the whole expert panel; and (6) further DELPHI rounds using the whole expert panel to follow until a consensus (and sufficient stability) was reached.

Accordingly, before applying the DELPHI survey, five panelists (the ‘core group’: CG, GC, NAD, CM, TT) conducted literature reviews to inform the generation of statements about TC and the TCP in general and specific to RBMCs. These statements were checked for duplicates, merged or reworded if applicable and the resulting 125 statements then provided to the whole expert panel to be scored (DELPHI Round 1).

**Fig. 1:**
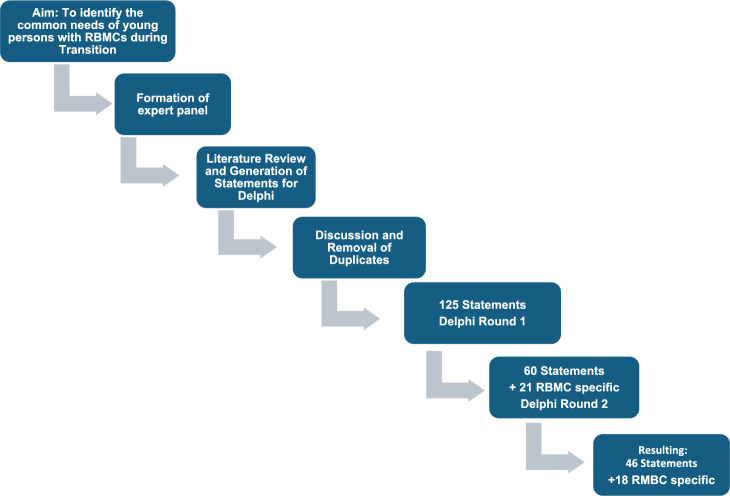
Generation and scoring of Statements in the Delphi Process. A consensus of at least 70% was chosen as the threshold to carry a statement forward to the next round. The full list of statements is in Table 1 and Table 2

No precondition was set on how many DELPHI rounds to follow. However, owing to the high rate of consensus and stability, it was decided that following the second round of statement scoring, no further rounds were necessary and that a workable number of priority statements had been derived. See Fig. 1 for the Delphi process.

## Results

A total of 125 statements were generated, which were reduced to 60 general and 21 RBMC-specific statements after elimination of duplicates and exclusion of low-scoring/dismissed statements (DELPHI Round 1) based on the Calibrum Tool scores. For workflow see Fig. 1.

The 60 + 21 statements (see Table 1 and Table 2 for full list) covered the following domains of action of the TCP: (1) Initiation and Start of TC; (2) Planning of TC; (3) Requirements of a successful TCP; (4) Empowerment of patients within the TCP; (5) Organization and communication within the TC service; (6) Service infrastructure and funding; and (7) Clinical care. RBMC-specific statements addressed different domains of action. All statements were submitted to the second round of the DELPHI process. All panelists responded to both rounds of the Delphi.

**Table 1:** General statements regarding the transition care (TC) that were entered into the DELPHI

ID	Item	Level of consensus, %	Status
		Cut-off ≥ 70%	
		First round	
Initiation of process			
1	Initiation of TC should be available for all young persons from the age of 14 years	**70,20**	**Agreement**
2	There is no set age at which TC should be started as long as it is early; e.g. from 12 years old onwards	56,29	Disagreement
3	TC should be discussed with young persons and their parents/guardian from the age of 12 years	60,96	Disagreement
4	TC should not be started before the age of 16 years	33,22	Disagreement
5	TC should be introduced at no specific age, but when pediatrician evaluation suggests readiness to transition	48,73	Disagreement
6	Initiation of TC is a joint decision of pediatrician, the young person and parents/guardian	59,94	Disagreement
7	TC should not start until the young person is emotionally prepared to leave Pediatric Service	41,69	Disagreement
8	The involvement of parents/guardian in deciding when to initiate TC is not important	36,10	Disagreement
9	TC should be initiated solely by the pediatric team	33,24	Disagreement
Start of the TC process			
10	A structured assessment of readiness for TC should always be done	**80,93**	**Agreement**
11	The goals of TC should be defined at the beginning of TC	**88,37**	**Agreement**
12	The young person/adolescent should be provided with information about their condition	**88,92**	**Agreement**
13	The TC pathway should be structured and explained at the beginning to the young person and their parents/guardian	**86,40**	**Agreement**
14	A structured TC pathway should be devised and agreed with the young person and their parents/guardian	**76,82**	**Agreement**
15	A TC plan should be developed initially with the young person	67,15	Disagreement
16	The pace and structure of TC should be planned involving the young person	**86,71**	**Agreement**
Planning of requirements			
17	There needs to be defined roles for all members of the core TC team	**88,67**	**Agreement**
18	A specialist nurse should always be the TC coordinator	58,55	Disagreement
19	A dedicated TC coordinator should be appointed and become point of contact for the young person	**87,17**	**Agreement**
20	The young person should meet their TC coordinator before leaving pediatric care	**84,86**	**Agreement**
21	There needs to be a direct line of contact by phone/email/text to a designated core TC team member	**79,61**	**Agreement**
22	The young person, their parents/guardian and the TC team all need to agree when TC is complete	69,55	Disagreement
Success of transition process			
23	Parents /guardians should be kept informed and involved in decisions throughout the TC period	**83,44**	**Agreement**
24	School, therapy and other supporting services should be notified of the change in point of contact to the designated core TC team member	**85,11**	**Agreement**
25	The presence of the parents/guardians at the start of the TC process is desirable	**79,09**	**Agreement**
26	The presence of the parents/guardians at the start of TC process is not mandatory	63,98	Disagreement
27	Aspects of the change in legal responsibilities should be explained to the young person and the parents/guardian	**84,29**	**Agreement**
28	Early preparation of the TC process includes education of the young person and the parents/guardians on the TC process	**86,39**	**Agreement**
29	TC should provide protected time for consultation between TC clinicians and the young person alone, as well as with their family/guardian	**84,15**	**Agreement**
Importance regarding empowerment			
30	In early TC the young person should be facilitated to learn about their rare condition, self-management, self-advocacy and 3rd sector support	**86,74**	**Agreement**
31	Before TC starts, a document detailing local arrangements for TC should be shared with the young person and their family/parents/guardian	**84,62**	**Agreement**
32	The young person should be educated about their condition, the treatment plan, and self-management skills	**89,86**	**Agreement**
33	In TC the young person should be provided with information on appointments, investigations, treatments, and how to communicate effectively with clinicians	**90,73**	**Agreement**
34	There should be a TC day for the young person and parents/guardian where aspects of education relevant to empowering the young person are delivered by the TC team	67,14	Disagreement
35	The TC team should provide information to the young person about local community and patient support groups	**87,50**	**Agreement**
36	The TC care team should encourage and facilitate the young person to develop a peer support network	**80,73**	**Agreement**
Organization and communication			
37	The TC service should have a written agreed, regularly updated TC policy/Standard Operating Procedures document	**90,17**	**Agreement**
38	School, Physio and other support organizations should be notified of change in point of contact	**81,46**	**Agreement**
39	Young persons and their families/parents/guardian should know who is point of contact for them, in the TC team, and how to contact that person	**93,02**	**Agreement**
40	TC for young persons with RBMCs should align with the 12 EULAR/PReS standards and recommendations for the transitional care of young persons with juvenile-onset rheumatic diseases	**72,41**	**Agreement**
41	Adult and Pediatric Services clinicians should decide case management responsibilities before the young person leaves pediatric care	**76,24**	**Agreement**
42	A meeting or consultation with the adult clinicians should be organized for the young person with their families/parents/guardian present	**73,04**	**Agreement**
43	Regular joint meetings (e.g. every 6 months) between the young person with the RBMC, the pediatrician and adult physician are the key to a successful TC	54,71	Disagreement
44	A plan for responsibilities through the TC process needs to be agreed between the young person and the TC team	**83,46**	**Agreement**
45	The frequency of TC contacts needs planning and individualizing	**77,19**	**Agreement**
46	Evaluation of the TC process should be done by the TC care team during the TC pathway (e.g. mid-way) and plans adapted, if needed, accordingly	**79,74**	**Agreement**
47	Face to face meetings with the young person should always be in an agreed age-appropriate environment	**77,70**	**Agreement**
48	There needs to be a direct contact facility for the young person, by phone/email/text, ideally with a specialist nurse	**77,86**	**Agreement**
Infrastructure and funding			
49	There should be secure funding for resources to provide uninterrupted TC for all young persons with rare bone conditions	**84,51**	**Agreement**
50	TC needs to be provided within a framework of social, religious and cultural norms relevant to the young person, and should exist consistent of any local, and the national, legal framework for health care	**83,46**	**Agreement**
51	It is desirable to have funding for uninterrupted clinical care during TC	**78,28**	**Agreement**
52	There must be secure funding for dedicated resources to provide uninterrupted TC Service for young persons, entering adult care environment	**82,49**	**Agreement**
53	Commissioners should include yearly measurement of aspects of TC Service performance, in judging TC Service success	**83,43**	**Agreement**
Clinical care			
54	TC plans should include planning to educate young persons on their condition, self-management skills, and how to advocate for themselves	**88,57**	**Agreement**
55	All TC plans should align with standards of care and/or guidelines (if they exist) for the young person’s rare condition	**88,17**	**Agreement**
56	PROM/PREMs should be used as part of TC, if they have been validated for the specific rare bone condition in question	**80,69**	**Agreement**
57	Multidisciplinary care should be utilized where possible	**74,98**	**Agreement**
58	During TC, young persons should be encouraged to contact and interact with the appropriate patient support group for their rare bone condition	**78,82**	**Agreement**
59	Young persons with a rare bone condition should be advised to use only credible sources of information to facilitate self-management and monitoring for any associated evolving comorbidities	**80,51**	**Agreement**
60	The TC care team should encourage and facilitate TC patients to develop a peer support network	**79,49**	**Agreement**

In bold are statements that reached the threshold of at least 70% and for which a consensus was achieved

**Table 2:** Statements for the TC process (TCP) specific for RBMC statements were accepted if at least 70% of consensus was reached

ID	Item	Level of consensus, %	Status
		Cut-off ≥ 70%	
		Second round	
Success of transition process			
1	In RBMCs it is essential to collect prior information on genetic and imaging results at start of the Transition Care (TC) process	**74,7**	**Agreement**
2	Depending on the type of RBMCs the TC team may consist of additional experts e.g. physiotherapist, dentist	**74,4**	**Agreement**
3	The type of RBMC may be differentiated according to the lead symptom(s) to inform the formation of the TC team and the future care team	**77,2**	**Agreement**
4	In planning TC, the need for, and identification of ‘specialists and experts relevant to the RBMC, should be initially discussed with the young person and their carers	**80,2**	**Agreement**
5	Young persons should be encouraged to contribute to TC planning by being informed by advice/ guidance from their RBMC patient-led organization	**76,5**	**Agreement**
6	Expectations of any treatment or surgery to be undertaken after leaving pediatric services needs to be made clear and an appropriate healthcare professional should be identified	**77,4**	**Agreement**
Organization and communication			
7	In RBMCs it is essential to communicate to the young person the different specialties/ specialists that/ who will be involved in the future care—but may be not necessarily be part of the TC team (e. g. ophthalmologist, dentist…)	**72,8**	**Agreement**
8	In RBMCs the team of specialists involved in future care needs to be individualized	**84,4**	**Agreement**
9	In RBMCs the team of specialists in future care often involve endocrinologists, rheumatologists, osteologists, physiotherapists, dentists, ophthalmologists, orthopedic surgeons, etc	**72,4**	**Agreement**
10	During TC it is important to discuss how responsibility for consent to allow access to personal data changes once they are 18 years old. This may arise specifically for some but not other RBMCs given the availability of specific registries for various condition	65,8	Disagreement
11	Young adults with OI, XLH and RBMCs manifest by skeletal fragility, should be encouraged to act as their own advocate in helping to transfer information between acute medical facilities/ hospitals and their TC coordinator, should they attend another hospital for sustaining a fracture	**75,0**	**Agreement**
12	Young persons should be told what to do in the event of fractures including when to seek treatment and where to go for appropriate treatment for the fracture as the location of treatment may vary depending on severity	**85,5**	**Agreement**
Infrastructure and funding			
13	It is suggested to organize TCs of young persons with RBMC to tertiary care settings if possible	**83,5**	**Agreement**
14	Outpatient clinics space, including waiting areas, should be sufficient to accommodate RBMC persons attending in wheelchairs	**80,0**	**Agreement**
15	Young persons should be signposted to relevant third sector organizations which can provide additional support after leaving pediatric services	69,9	Disagreement
16	As part of TC, young persons should be directed to peer groups, in person or virtually through social media	**78,9**	**Agreement**
Clinical care			
17	During TC the young person should become familiar with genetic results (if available)	**86,2**	**Agreement**
18	Counseling regarding pregnancy, birth and family planning in RBMCs should be offered/ organized during TC	**84,3**	**Agreement**
19	If not considered prior to TC, advice on contraception and clinical genetics advice on heritability of genotype, should be considered appropriate during TC, after the age of 16 years	**84,4**	**Agreement**
20	TC plans should include details of procedures for the management of fractures or other clinical complications where they differ to that of pediatric care	69,6	Disagreement
21	Part of TC should include education young persons of the associated comorbidities of their condition and the symptoms they need to look out for	**79,3**	**Agreement**

In bold are statements that reached the threshold of at least 70% and for which a consensus was achieved

Recommendations for the TCP in RBMC are shown in Table 1 and Table 2 and described in the domains of action, in the following paragraphs:

(I) Initiation and start of TC
  The timing of the initiation of transitioning, for a young person with a RBMC, should be a joint decision between the young person with the condition, the family/guardian and the RBMC healthcare team. A TCP should be available for all young persons from the age of 14 years. However, the actual initiation of TCP should follow an agreement that the young person has emotional readiness for the process.
(II) Planning of TC
  At the beginning of the TCP the goals of the TCP should be clearly defined, and the young person and the family/guardian should be provided with information not only about the health condition but also about the TCP itself. Accordingly, the pace and structure of the TCP should be agreed upon by the young person, the family/guardians and the healthcare professionals.
  Early preparation includes education of the young person and the parents/guardians on the TCP. The presence of the family/guardians at the start of the TCP is therefore desirable and they should be kept informed and involved in decisions throughout the TCP. However, during the TCP protected time for consultation between TC clinicians and the young person alone should also be provided.
(III) Requirements of a successful TCP
  It is advisable to identify and assign roles to the healthcare professionals within the transition care team (TCT). The role of a transition care coordinator (TCC) should be assigned specifically and the young person with the RBMC should meet with the TCC before leaving pediatric care and should be given a direct, initial point of contact route to the TCC using phone, email or text.
  Education forms the bedrock of empowerment in TC extending beyond the condition itself to encompass a comprehensive understanding of the treatment plan and essential self-management skills. Information dissemination during TC should cover pivotal aspects such as appointments, investigations, treatments, and effective communication with clinicians.
  An evaluation of the TCP should be undertaken by the TCT during TC (e.g. mid-way) and plans should be adapted, accordingly.
(IV) Empowerment of patients within the TCP
  At the onset of TC, there is a collective recognition that the young persons must gain awareness of their RBMC and be actively empowered to cultivate self-management skills, advocate for themselves, and tap into the valuable support offered by the third sector. Prior to embarking on the TCP, transparency about the process is key. Accordingly, a comprehensive document outlining local arrangements should be shared with both the young person and their family/parents/guardian/caregivers.
  A holistic approach to empowerment should consider providing specific *transition care days* for young persons and their parents/guardians, to facilitate knowledge transfer of the RBMC and the relevant care process.
  The TC team (TCT) should assume a pivotal role in connecting young persons with local community and patient support groups, creating a network that empowers through shared experiences. The facilitation of a peer support network is central to the TCT’s mission.
  The key changes in legal responsibilities should be explained to the young person with The RBMC and the parents/guardian. School, therapy and other supporting services should be notified of the change in point of contact to the designated core TCT member.
(V) Organization and communication within the TC service
  A plan for responsibilities through the TCP needs to be agreed on between the young person and the TCT. This TC plan should be updated regularly.
  Face-to-face meetings with the young person should always take place in an agreed, age-appropriate environment.
  Before leaving pediatric care, a meeting or consultation with the adult clinicians should be organized for the young person with their families/parents/guardian present. There needs to be a direct contact facility for the young person, by phone/email/text, ideally with a specialist nurse. Case management responsibilities need to be clarified before the young person leaves pediatric care.
(VI) Service infrastructure and funding
  Securing funding for resources to provide uninterrupted TC for all young persons with an RBMC is considered necessary. Resources should be provided to include yearly measurement of aspects of TC and service performance.
  TC needs to be provided within a framework of social, religious and cultural norms relevant to the young person and should be provided cognizant of any local, and the national, legal framework for health care.
(VII) Clinical care
  In general, TC plans should include how to educate young persons on their condition, self-management skills, and how to advocate for themselves. In addition, TC plans should align with standards of care and/or guidelines (if they exist) for the young person’s RBMC.
  Young persons with RBMCs in TC should be supported to learn self-management techniques, advocate for themselves and to interact with peer support organizations.

### Organization of Care Specific to RBMCs

Specific modifications of all domains of action of the TCP are suggested for young persons with RBMCs (see Table 3).

A. RBMC-specific features (e.g. joint stiffness, short stature, bone fragility, endocrine pathology etc., should be identified and an agreement on suggested frequency of visits and reasons for visits (condition selective) decided.
B. RBMC specific, validated or accepted clinical and or radiological scoring systems should be employed where possible (e.g. for joint stiffness, pain).
C. It is of utmost importance to identify the most relevant RBMC-specific adult care specialist.
D. A multidisciplinary TCT, ideally expert in the RBMC, including an occupational therapist and physiotherapist is mandatory. A full TCT may include further subspecialty experts e.g. Dentistry, ENT, Ophthalmology, Neurosurgery, Orthopedics, Rheumatology, Osteology and Endocrinology.
E. Data access across health care providers or transfer of relevant results/information needs to be ensured, especially radiographic images, bone density data, genetic results, and information on counseling regarding family planning/ mode of inheritance of the condition.
F. Information of contact points for emergency care and information of existent referral centers in specific countries and at the European level and the European reference networks (ERN BOND, ENDO ERN) should be mentioned.

**Table 3:** Steps of the transition process and suggested adaptions for RMBCs

	Process step	Agreement	Modification in RBMCs	Example
I	Initiation and start of the TC	– TC should be available from the age of 14 years	No adaptations for RBMC	NA
		– Transition readiness should be assessed		
		– TC should be planned and structured, and the plan should be assessable and modifiable		
		– The young person should be actively involved in the planning of TC		
II	Planning of requirements	– Each member of the TC team should have a clear and defined role	– Identify current and potential lead symptoms	e.g. short stature in skeletal dysplasia, fractures in osteogenesis imperfecta, Joint stiffness in XLH, endocrine condition in pseudohypoparathyroidism/ Inactivating PTH/PTHrP signaling disorders
		– A designated TC coordinator should be the primary contact for the young person		
		– A direct line of contact (e.g. via phone, email or text) is recommended		
		– The TC coordinator and the young person should meet before the young person leaves pediatric care		
III	Requirements for success of transition process	– Parents/guardians should be involved in decisions, especially at the start of TC	– Agree on monitoring/ therapy goals for lead symptoms	e.g. dental specialist in XLH; ENT in hyperostotic conditions, orthopedic surgeon in skeletal dysplasia
		– Schools and support services can be informed of the new TC contact	– The TC team should gather genetic and imaging data	
		– The young person and their family should be educated about TC early, and aspects of the change in legal responsibilities should be explained	– The TC team should include physiotherapist and occupational therapist and may include additional specialists	
		– TC should provide dedicated time allocated for private consultations of the young person with clinicians	– Expectations for any treatment or surgery after leaving pediatric services should be clarified	
IV	Empowerment	– In early TC the young person should be supported in learning about their condition, self-management and 3rd sector support	– Ensure contact to patient organization	e.g. local groups, https://ernbond.eu/; https://endo-ern.eu/mtg-2-overview/;
		– A document outlining local TC arrangements should be provided to the young persons and their family	– Ensure information on respective European reference network (ERN) is provided (ERN BOND, ENDO ERN)	Rare Bone Disease Alliance (2019). Guidelines for Empowerment and Self-management
		– The TC team should offer information on appointments, treatments and effective communication with clinicians		
		– The TC team should support connection of the young person with patient support groups		
V	Organization and communication	– The TC team should establish an individualized care plan, including the frequency of contact and regular evaluations to adapt plans if needed	– Agree on frequency and reason for visits, use scoring system if possible	e.g. apply mobility score, rickets score, fracture risk assessment, etc
		– Support services (e.g. schools, physio) should also be notified of contact changes	– TC for young persons with RBMCs should align with EULAR/PReS standards, and the TC service should have an updated policy document	
		– Meetings with the young person should occur in age-appropriate settings	– RBMC teams often include endocrinologists, rheumatologists, osteologists, physiotherapists, dentists etc	
		– A meeting with adult-care clinicians can be arranged for the young person and their family, a	– Young adults with conditions like OI or XLH should be encouraged to advocate for themselves, especially in emergency situations like fractures, and be aware of appropriate treatment protocols	
		– The adult-care and pediatric-care clinicians should decide case management responsibilities		
		– The young person should be informed about the range of specialists involved in their future care		
VI	Infrastructure and funding	– TC teams should secure funding to ensure consistent, uninterrupted service	– Young persons should be connected to 3rd sector organizations and peer groups	Specialized Services: Dental, ENT, Ophthalmology, Neurosurgery, Orthopedics, Rheumatology, Osteology, Endocrinology, Genetics
		– TC should align with the young person’s social, cultural, and religious context, as well as national health laws	– Outpatient clinics space, including waiting areas, should be sufficient to accommodate RBMC persons attending in wheelchairs	
		– Performance of TC services should be reviewed annually		
		– Therapists and Social Workers should be part of the TC team if necessary		
VII	Clinical care	– TC plans should align with relevant care standards and guidelines (if they exist)	– Transfer relevant results/ information to adult care	Radiographic images, Bone Density results, Genetic results, Counseling re family planning/inheritance
		– PROMs/PREMs should be integrated into TC	– TC plans should focus on educating young people with RBMCs about their diagnosis, self-management, and self-advocacy skills	
		– Multidisciplinary care should be utilized		
		– TC should include genetic counseling, education on related comorbidities, and guidance on family planning, including contraception and genetics, for those over 16		

## Discussion

With this work, the Rare Bone Disease action group of ECTS provides a DELPHI consensus on TC for young persons with a RBMC. This Delphi consensus reflects expert opinion and offers an important first step toward standardized transition care in RBMCs. Future empirical studies are needed to assess the real-world impact and feasibility of these recommendations.

To our knowledge, these are the first recommendations for RBMC TC services. While there are some expert consensus recommendations for specific RBMCs e.g. for osteogenesis imperfecta [17] and XLH [14], there are none which provide guidance for Departments and Services tasked with managing persons with a spectrum of different RBMCs. Of note, the consensus recommendations presented here align with key transition care principles described in the Asia–Pacific consensus on XLH [14] and the EULAR/PReS recommendations for juvenile-onset rheumatic diseases [15]. However, our work specifically addresses the complexity and variability of RBMC, emphasizing individualized timing, multidisciplinary input, and condition-specific challenges not fully captured in these prior frameworks.

We originally opted against conducting a survey on current TC practices as done by Casado et al. [11] and Shishkov et al. [21] as TC is underfunded in most countries [22] and thus current practice will represent local solutions that are highly dependent on the interest and motivation of individual clinicians, the size of the Hospital Department unit available resources.

One of the crucial points in undertaking a Delphi survey lies in the selection of the expert panel members who participate in the anonymous and non-anonymous voting process of the Delphi [19, 23]. Accordingly, we have combined patient representation of three major RBMCs, and clinical experts (> 10 years of clinical experience in the field) from different disciplines from pediatric and adult care and representing different European countries.

Despite the heterogeneity of background, the panelists, there was high consensus on most statements that were generated, indicating that experts and patient representatives had a similar understanding of the requirements of TC.

The consensus derived for TC for young persons with RBMCs here, importantly reflects the recommendations of the twelve European League Against Rheumatism (EULAR) standards and recommendations for the TC of young persons with juvenile-onset rheumatic diseases [15].

We, like others, have concluded that effective TC should ensure both continuity of medical management and address the unique psychosocial aspects associated with chronic rare diseases. For young persons with RBMCs, timely initiation of the TCP, starting as early as by age 12–14, or when transition readiness is established [24], has been shown to improve readiness for transfer to adult services and decrease potential gaps in care. Several validated tools, such as the Transition Readiness Assessment Questionnaire (TRAQ) [24] and the web-based toolbox ‘Got Transition’s Six Core Elements of Health Care Transition™ ‘ (Got Transition Resources) offer structured approaches to transition, and customizable tools for assessing readiness, planning, and transfer of care. While not developed specifically for RBMCs, they can be adapted to this population with condition-specific modifications. Implementing a structured transition with clear role assignments within the TCT helps optimize communication between pediatric and adult providers and facilitates individualized care planning [25, 26].

These consensus recommendations have highlighted the importance of fostering empowerment of the young person with a RBMC, which is a dynamic process encompassing education, self-advocacy, and community integration. These elements collectively empower young persons as they navigate the complexities of these rare conditions and have been described previously for other rare diseases as well [8, 25].

Providing access to peer support networks and resources is particularly effective in enhancing resilience and confidence during the TCP [27]. However, it should be mentioned that peer support groups are often led by older adults and may not have a young person’s group, which may limit the attractiveness of patient groups to adolescents and commonly have limited representation of ethical minorities which may limit the group's attractiveness to younger participants from various backgrounds.

Despite the agreement on many aspects of the TCP, the variability in healthcare systems across Europe will challenge any uniform implementation of TC guidelines [5, 28]. Our consensus recommendations underline the importance of integrating legal, cultural, and financial considerations specific to each country into the TC framework. For example, disparities in funding and availability of transition-specific resources may limit the consistency of care across regions, suggesting a need for flexible but standardized TCP frameworks to bridge these differences. Particular challenges exist in low-resource healthcare systems, where infrastructure and funding constraints may limit implementation. Approaches such as stepwise integration, use of digital platforms, or adaptation of essential elements of care may enhance feasibility. Barriers to implementation may also include lack of formal reimbursement for transition care coordination roles, limited institutional support for cross-specialty meetings, and low levels of engagement from adolescents or families unfamiliar with long-term care planning. Identifying scalable, sustainable models is critical for broader implementation. Based on the consensus process, we suggest that transition programs for RBMCs should aim to implement a minimum standard of care regardless of local resources or specific diagnosis. This includes: A designated transition coordinator or contact person and an individualized written care plan shared with the adult team. These elements could serve as measurable benchmarks across diverse healthcare settings.

## Limitations of the Study

The Delphi consensus recommendations study is inherently limited by the subjective nature of expert opinion, which may not fully capture the diverse experiences of young persons with RBMCs across different healthcare settings. We acknowledge that this expert panel primarily included participants from European countries and medical disciplines already engaged with the ECTS Rare Bone Disease Action Group. While this enhances alignment with current clinical practice in Europe, it may limit the generalizability of the findings. Broader international involvement is encouraged in future efforts.

Additionally, the study's focus on selected conditions may limit the generalizability of the findings to other rare bone conditions. Variability in healthcare resources and systems across Europe further restrict the ability to apply these recommendations. Finally, while efforts were made to include patient representatives, the absence of real-world testing of the recommendations means that the practical feasibility of the recommendations remains to be validated.

## Strength of the Study

Despite the high relevance of TC in young persons with RMBCs there is paucity of studies and especially data to guide our TC clinics. This Delphi method allows for a consensus build from patient advocates and many medical specialists of different disciplines and includes a set of clear suggestions for improvement. RBMCs encompass a wide range of heterogeneous disorders with varying disease trajectories, treatment requirements, and psychosocial impacts. While this variability allows for a uniform transition model as a base, it also requires flexible, individualized approaches.
